# Nativity and Serum Concentrations of Antioxidants in Mexican American Children: A Cross-Sectional Study

**DOI:** 10.3390/nu6041598

**Published:** 2014-04-16

**Authors:** Kamal Eldeirawi, Mary Dawn Koenig, Victoria Persky, Noel Chavez

**Affiliations:** 1 Department of Health Systems Science, College of Nursing, University of Illinois at Chicago 845 S. Damen Ave. Room 1054 (MC 802), Chicago, IL 60612, USA; 2 Department of Women, Children and Family Health Science, College of Nursing, University of Illinois at Chicago 845 S. Damen Ave., Room 814 (MC802), Chicago, IL 60612, USA; E-Mail: marydh@uic.edu; 3 Division of Epidemiology and Biostatistics, School of Public Health, University of Illinois at Chicago1603 W. Taylor St., 877 SPH-PI (MC 923), Chicago, IL 60612, USA; E-Mail: vwpersky@uic.edu; 4 Division of Community Health Sciences, School of Public Health, University of Illinois at Chicago 1603 W. Taylor St., 659 SPH-PI (MC 923), Chicago, IL 60612, USA; E-Mail: nchavez@uic.edu

**Keywords:** Mexican Americans, nativity, acculturation, immigration, antioxidants, carotenoids

## Abstract

There is limited research on the effect of immigration on biological markers of nutrition among children of Mexican origin in the United States. The purpose of this cross-sectional study was to examine data from the Third National Health and Nutrition Examination Survey (NHANES III) (1988–1994), on a national and representative sample of 1559 Mexican American children, 4–16 years of age, and assess the associations of country of birth with serum concentrations of carotenoids, vitamin A, and vitamin E. In multiple regression analyses, Mexico-born Mexican American children had significantly higher serum concentrations of α-carotene, β-carotene, β-cryptoxanthin, lutein/zeaxanthin, vitamin A, and vitamin E than their counterparts who were born in the United States after adjustment for age, sex, poverty income ratio, level of education of family reference person, body mass index, total serum cholesterol, serum cotinine, total energy intake, and vitamin/mineral consumption. Our findings confirm evidence for a negative effect of immigration/acculturation on dietary quality in this population. These findings also suggest that immigrant Mexican families should be encouraged to maintain their consumption of fruits and vegetables. Prospective studies are needed to further assess the effects of immigration/acculturation on diet and other health outcomes in children of Mexican origin and immigrants.

## 1. Introduction

Hispanics constitute the largest and one of the fastest growing ethnic populations in the United States [1]. The approximately 52 million U.S. Hispanics represent 17% of the country’s population [2] and are projected to account for nearly one-third of the U.S. population in 2060 [3]. In 2012, 24% of U.S. children were Hispanic and the percentage of Hispanic children in the U.S. increased substantially in the last few decades while most other race and ethnic groups in the U.S. experienced a decline in the percentages of children [4]. Roughly two-thirds of Hispanics in the country are of Mexican origin [2]; they comprise around 10% of the U.S. population, and experienced rapid growth rates with an increase of 11.2 million between 2000 and 2010 (63% due to births) [1]. Immigrants continue to account for a large percentage (39% or 12.4 million) of Mexican Americans (MAs) in the U.S. [5]. Nonetheless, MAs remain a very understudied population and the impact of the transition related to the immigration and acculturation experiences on MAs’ health conditions and behaviors is not clearly understood. In addition, less is known about MA children with regard to the nutrition transition. 

Mexican American adults and children are at increased risk of chronic conditions that have been linked with nutritional status. Mexican American adults experience higher prevalence rates of diabetes [6,7], obesity [8,9,10], and heart disease related outcomes [9] than their non-Hispanic white counterparts. In the multi-ethnic participants in the Study of Latinos, MA men had the highest while MA women had the second highest diabetes prevalence among the varied Hispanic ethnicities, 19.3% and 18.5%, respectively [11]. 

Mexican American children also are more likely to be at risk for overweight and obesity [8,12] as well as diabetes [13] than their non-Hispanic white peers. In U.S. children without a history of diabetes, MA children had higher levels of hemoglobin A 1c (which has been used to screen for diabetes [14]) than non-Hispanic white children [15]. Studies also suggest a rapid increase in the incidence and prevalence rates of type 2 diabetes in Hispanic children [16,17]. 

The transition MA children and adults experience through the processes of acculturation and immigration has been linked with several health outcomes and behaviors, including overweight/obesity, diabetes, cardiovascular disease, and asthma as well as other respiratory conditions [11,15,18,19,20,21,22,23,24]. Reasons for this change in chronic disease risk profile as MAs become more acculturated in the U.S. are not well understood. Nonetheless, one potential mechanism to explain the negative impact of acculturation or immigration on health is the often unhealthful changes in food intake and resulting nutritional status that accompany Mexican immigrants’ transition to the U.S. mainstream culture [25,26,27]. Studies in adults have documented significant declines in MA’s consumption of fruits and vegetables with acculturation. Mexican American adults born in Mexico consumed less fat and more fiber; vitamins A, C, E, and B6; folate; calcium; potassium; and magnesium than their counterparts born in the U.S. [28]. In another study, first generation MA women had significantly greater intake of protein, carbohydrates, cholesterol, vitamins A and C, folic acid, and calcium than second generation MA or white non-Hispanic women [29]. Other studies found an inverse association between length of residence in the U.S. among Mexican women and consumption of vitamin A-rich and vitamin C-rich fruits and vegetables [30]. Evidence based on data from NHANES III [27] shows that Mexican-born adults have the highest serum levels of α-carotene, β-carotene, β-cryptoxanthin, lutein/zeaxanthin, and total carotene compared with their peers born in the U.S. or other countries. These studies show the importance of examining within group differences to help understand the changes that immigration and resultant acculturation have on health and nutrition status. 

There is limited research on dietary transition in MA children and it is not clear if or how their nutritional status is affected by immigration or acculturation differently than adults [24]. Our analysis of dietary data from NHANES III documented significant variations in dietary intakes among MA children by nativity [15]. Another NHANES analysis (1999–2004) suggested that acculturation was associated with poorer dietary quality in MA adolescents 12–19 years of age with second and third generation MA consuming less fruit, whole fruit, vegetables, grains, and meats but more sweetened beverages, whole grains, saturated fat, sodium, oil and energy from discretionary foods than Mexican-born youth [24]. Most studies linking acculturation or nativity with lower dietary quality in MAs have been based on research with adults and studies on the effect of immigration on dietary changes in MA children remain scarce. In addition, much of this limited research has been based on dietary recalls rather than objectively measured biological nutritional status markers in MA children. This study used data from NHANES III to examine the associations of serum concentrations of the carotenoids, vitamin A, and vitamin E with country of birth in a nationally representative sample of U.S. MA children and adolescents.

## 2. Experimental Section

### 2.1. Design and Participants

This cross-sectional study is based on data from NHANES III, which was conducted 1988–1994. The survey utilized a complex, multistage, probability sampling design to select a sample representative of the U.S. non-institutionalized civilian population age 2 months or older. The survey oversampled MAs, African Americans, and young children (2 months-5 years of age). In NHANES III, 2236 U.S born and Mexico-born participants 4–16 years of age had complete data on serum concentrations of α-carotene, β-carotene, β-cryptoxanthin, lutein/zeaxanthin, and lycopene and vitamins A and E (α-tocopherol). We truncated the extreme outliers by excluding the observations with the lowest or largest 0.5% of the serum concentrations [31]. Therefore, the sample size was reduced to 2126 participants. Of those, data were missing on educational level of the family reference person (*n =* 26), poverty income ratio (*n =* 236), body mass index (BMI) (*n =* 34), serum total cholesterol (*n =* 2), serum cotinine (*n =* 218), total caloric intake (*n =* 117), and consumption of vitamins/minerals in the past month (*n =* 18). The final sample size used in this study was 1559 participants with complete data on all the variables included in the current analyses. The Institutional Review Board of the University of Illinois at Chicago has determined that this research is exempt because it utilized publicly available data with no personal identifiers. 

### 2.2. Variables

Participant serum samples were analyzed for a variety of biological markers of nutrition including five carotenoids (α-carotene, β-carotene, β-cryptoxanthin, lutein/zeaxanthin, and lycopene), vitamin A, and vitamin E (α-tocopherol). Serum concentrations of these nutritional biomarkers (ug/dL) were analyzed using reversed-phase high performance liquid chromatography. This procedure requires a long time to analyze lutein and zeaxanthin separately. Therefore, serum concentrations of lutein and zeaxanthin were measured together and combined in one single value as published in the NHANES dataset. 

The main explanatory variable examined in this analysis was country of birth (U.S. *vs*. Mexico) as reported by parents/guardian of participant children. Although there is no gold standard measure of acculturation, country of birth is a very commonly used proxy measure of acculturation and is highly correlated with other indicators of acculturation such as language use [32,33]. 

The control variables used in this analysis included age, gender, educational level of family reference person, poverty income ratio (defined as the ratio of the family’s income to the families appropriate poverty threshold as determined by the U.S. Census Bureau), BMI defined as weight in kilograms divided by height in meters squared, serum total cholesterol (mg/dL), serum cotinine (ng/mL), and use of supplemental vitamins/minerals in the past month (yes *vs*. no). All these covariates, except for gender and use of supplements, were treated as continuous variables and were centered around the mean in multiple linear regression analyses. 

### 2.3. Statistical Analysis

Statistical analyses were conducted using SAS version 9.3 (SAS Institute, Cary, NC, USA) incorporating sampling weights to provide estimates adjusted for the complex sampling design used in NHANES. We used linear regression models to assess the associations of country of birth and the dependent variables (serum concentrations of antioxidants) as well as continuous covariates. Serum concentrations of antioxidants were first regressed individually on country of birth to provide estimates of the crude associations of country of birth with the nutritional biomarkers. Then separate multiple linear adjusted regression models were conducted for each of the dependent variables with final models including country of birth, gender, age, use of vitamins and minerals in the past month, age, level of education of family reference person, poverty income ratio, BMI, serum total cholesterol, serum cotinine, total caloric intake. Chi-square statistics were used to compare U.S. and Mexico-born participants on categorical covariates (gender and vitamin/supplement usage in the past month). A *p* value of <0.5 was used to indicate statistical significance. 

## 3. Results

Characteristics of the study population and how they vary by country of birth are presented in Table 1. Of participants, 13.44% were born in Mexico, 50.66% were males and the average age was 10.57 years. U.S. born children were significantly younger, had a significantly higher poverty income ratio, had more educated parents/guardians, and were significantly more likely to take supplemental vitamins/minerals in the month prior to the survey than their Mexico-born counterparts.

**Table 1:** Characteristics of the Study Population.

Variable	All (*n =* 1559)		U.S. born (*n =* 1355)		Mexico-born (*n =* 204)		*p* value
	Mean	SE	Mean	SE	Mean	SE	
Age (years)	10.57	0.11	10.43	0.12	11.49	0.30	0.0010
Level of education of family reference person	8.97	0.14	9.37	0.15	6.37	0.34	<0.0001
Poverty income ratio	1.41	0.038	1.50	0.04	0.85	0.06	<0.0001
Body mass index (kg/m^2^)	19.69	0.14	19.60	0.15	20.26	0.42	0.1434
Serum cholesterol (mg/dL)	162.35	0.93	162.70	0.97	160.08	2.92	0.3948
Serum cotinine (ng/mL)	2.30	0.53	2.46	0.61	1.32	0.62	0.1912
Total energy intake (Kcal)	2013.94	29.67	2028.24	32.96	1921.92	57.35	0.1080
Male gender	50.66	1.69	51.14	1.82	47.56	4.47	0.4561
Took vitamins/minerals in the past month	24.14	1.49	25.26	1.64	16.91	3.29	0.0398

Table 2 includes the results based on logistic regression analyses. In crude linear regression analyses, compared with Mexico-born children, serum concentrations of α-carotene, β-carotene, β-cryptoxanthin, lutein/zeaxanthin and vitamin A were significantly lower while serum levels of lycopene were significantly higher in U.S. born MA children. 

**Table 2:** Crude Associations of Country of Birth with Serum Concentrations.

Independent Variables *	Intercept (mean for Mexico-born)	US-Born	SE	*p* value
α-Carotene	4.30	−0.62	0.22	<0.0040
β-Carotene	17.78	−2.09	0.82	0.0106
β-Cryptoxanthin	17.2	−3.83	0.78	<0.0001
Lutein/zeaxanthin	21.54	−3.13	0.80	<0.0001
Lycopene	18.94	2.45	0.85	0.0038
Vitamin A	39.64	−1.81	0.86	0.0351
Vitamin E	776.65	−16.64	17.71	0.3475

* ug/dL.

When linear regression models were adjusted for age, gender, educational level of family reference person, poverty income ratio, BMI, serum total cholesterol, serum cotinine, total caloric intake, and consumption of vitamins/supplements in the past month, mean serum concentrations of α-Carotene, β-Carotene, β-Cryptoxanthin, Lutein/zeaxanthin, vitamin A, and vitamin E were significantly lower in U.S.-born children than their Mexico-born peers (Table 3). Serum levels of lycopene did not vary significantly by country of birth. 

**Table 3:** Adjusted * Regression Coefficients for the Association of Country of Birth with Serum Concentrations.

Independent Variables **	Intercept	US-Born	SE	*p* value
α-Carotene	4.59	−1.03	0.21	<0.0001
β-Carotene	18.13	−3.27	0.84	0.0001
β-Cryptoxanthin	17.2	−3.79	0.75	<0.0001
Lutein/zeaxanthin	21.76	−3.754	0.75	<0.0001
Lycopene	19.99	1.15	0.84	0.1713
Vitamin A	38.66	−1.59	0.72	0.0263
Vitamin E	782.36	−43.15	12.81	0.0008

* Adjusted for age, gender, educational level of family reference person, poverty income ratio, BMI, serum total cholesterol, serum cotinine, total caloric intake, and consumption of vitamins/supplements in the past month; ** ug/dL.

## 4. Discussion

This study represents the first to address associations of immigration/nativity and serum levels of carotenoids and selected vitamins as objective measures of dietary intake in MA children. Compared to U.S. born MA children, those born in Mexico had lower socioeconomic status and were less likely to use supplemental vitamins. They also had significantly higher serum levels of carotenoids, and vitamins A and E. after accounting for known covariates. In the NHANES III children, MA children and adolescents had higher serum concentrations of α-carotene, β-carotene, β-cryptoxanthin and lower serum levels of lycopene than white children and adolescents [34]. However, while Ford and colleagues (2002) examined carotenoid levels in children and adolescents, they did not examine variations in MA children by country of birth [34]. 

Our results are consistent with studies suggesting negative impact of acculturation and immigration on not only dietary practices, but also biomarkers of dietary status. A study by Neuhouser and colleagues (2004) found highly acculturated MAs consumed fewer servings of fruits and vegetables per day compared with those not highly acculturated [35]. Duffey and colleagues (2008) also found that speaking Spanish was associated with a greater consumption of fruits among 3375 Mexican and 622 other Hispanic adults 18 years of age and older [36]. Our findings on MA children from NHANES III are also consistent with those investigating MA adult participants from NHANES. Stimpson and colleagues (2007) utilized data from NHANES III and showed that U.S. acculturation was associated with lower fruit and vegetable intake, as measured by carotenoid levels [27]. Those born in Mexico had the highest values of carotenoids, except lycopene [27]. The longer the individual lived in the U.S. the lower the carotenoid value in the individual [27]. In our study, we were not able to examine the impact of length of residence in the U.S. on the nutritional biomarkers because data were not available in the NHANES III dataset. 

Our study has some limitations. The study is based on cross-sectional data and therefore could not be used to establish a causal relationship between immigration and dietary quality. Although our study utilized data collected between 1988 and 1994, NHANES III remains the most comprehensive and nationally representative data of the U.S. population. We included data from NHANES III to avoid inconsistencies in the methods used to analyze blood samples for carotenoids between NHANES III and more recent collections of NHANES data. 

Strengths of the current study include its focus on an understudied population utilizing a nationally representative sample of MA children and using biological markers of antioxidants as opposed to relying on dietary recalls with their inherent measurement issues. This research does show the need to consider within ethnicity differences, MA in this case, when examining broader health risks and subsequent interventions or recommendations. 

## 5. Conclusions

Public health programs should encourage Mexican immigrants to maintain their healthy eating habits, including maintaining their traditional dietary practices, such as a high intake of fruits and vegetables and eating bread and potatoes without added fat. Identification of immigration-related changes, such as a modifiable factor like diet, may enhance our understanding of the etiology of many chronic conditions affecting children. More research is needed among MA sub-groups and stages of acculturation on dietary intake and biomarkers of dietary status. Additionally, studies examining dietary intake and biomarkers of dietary status across the U.S. and Mexico are needed. Findings from this study may improve and guide prevention programs aimed at reducing chronic diseases in children as well as adults.

One element missing from most research exploring disparities in health outcomes among ethnic/racial groups and the dominant culture is examining differences within the particular subpopulations. In this case, examining serum carotenoids and selected vitamin levels in MA children by birthplace/nativity shows the importance of not generalizing recommendations for all MA children. 
